# Penile Metastasis as the Presenting Symptom of Colorectal Carcinoma: A Rare Case Report

**DOI:** 10.1155/criu/8856762

**Published:** 2025-05-26

**Authors:** Mann Patel, Alain Kaldany, Farida Tanko, Andrew Parrott, Thomas L. Jang

**Affiliations:** ^1^Department of Urologic Oncology, Rutgers Cancer Institute of New Jersey and Rutgers Robert Wood Johnson Medical School, New Brunswick, New Jersey, USA; ^2^Department of Pathology and Laboratory Medicine, Robert Wood Johnson University Hospital, New Brunswick, New Jersey, USA

## Abstract

**Background:** Secondary penile cancer, despite the region's rich vascularization, is a rare phenomenon with only around 500 cases ever reported, typically of genitourinary origin and in even rarer cases, of colorectal adenocarcinoma. Unfortunately, the underlying mechanisms are not well elucidated, and prognosis remains poor with a late onset and median overall survival of 9 months for colon adenocarcinoma. Secondary penile cancer often presents alongside concurrent metastases months or years following successful treatment of the primary tumor. However, we report a case of an isolated penile metastasis as the presenting symptom of colon adenocarcinoma with no identifiable primary lesion or history of malignancy.

**Case Summary:** A 67-year-old African-American male presented with a 1-month history of voiding symptoms, whereupon follow-up revealed a penile mass near the left base of the penis. Postoperative histopathological, immunohistochemical, and genomic analyses revealed characteristics of invasive and metastatic colorectal adenocarcinoma. Initial diagnostic testing revealed elevations in serum tumor markers CA 19-9 and carcinoembryonic antigen, while whole body PET/CT scan and colonoscopy failed to identify any tumorigenic lesions or primary colorectal malignancy. Although hypermetabolic activity was noted near the base of the penis and bilateral inguinal lymph nodes, the patient is currently on chemotherapy with a modified FOLFOX-6 regimen with active surveillance and no adverse effects.

**Conclusion:** Here, we report a rare case of isolated penile metastasis as the first presentation of colon adenocarcinoma with no primary lesion. Regardless of origin, secondary penile cancer is a rare phenomenon with a poor prognosis. While the exact mechanism of spread is uncertain, the most probable mode of dissemination is through venous networks. There is also no standard of treatment relying on surgical, therapeutic, and palliative management. Although unclear, our unique presentation may portend a more favorable prognosis with continued treatment and observation.

## 1. Introduction

Penile metastasis, alternately referred to as secondary penile cancer, is a rare phenomenon, with only around 500 cases reported since 1870 [1]. The most common sites that metastasize to the penis include the bladder and prostate, with the lower gastrointestinal tract making up only around 80 cases in the literature [1, 2]. The typical age of onset of penile metastasis ranges from 60 to 80 years, mirroring the age of onset of the primary malignancy [1, 2]. Patients often present with obstructive symptoms including voiding difficulties, as well as penile or perineal pain [1–5].

Though it is rare, secondary penile cancer is often lethal, as it indicates worsened and disseminated primary disease [1, 2, 4]. In cases of rectal carcinoma metastatic to the penis, studies report an average overall survival of only 7 months, with patients presenting an average of 37 months following successful treatment of the initial disease [4]. Penile metastases from the colon are even fewer in number, despite colorectal cancer being the third most common cancer diagnosis and the second leading cause of cancer-related death in the United States (US) [3, 6]. While roughly one-half of patients with colorectal cancer will eventually develop disseminated disease, the typical sites of metastasis are the liver, thorax, and peritoneum [6, 7]. Interestingly, however, despite its relative proximity and extensive vascularity, penile metastasis of colorectal cancer remains a rare phenomenon [1, 2].

In most cases, secondary penile cancer presents alongside systemic metastases, which often appear months or years following successful treatment of the primary tumor [1–5, 8]. However, here, we report an unusual case of isolated penile metastasis as the initial presenting symptom of colon adenocarcinoma in the absence of a known primary colorectal lesion [3, 9, 10].

## 2. Case Presentation

**Figure 1:**
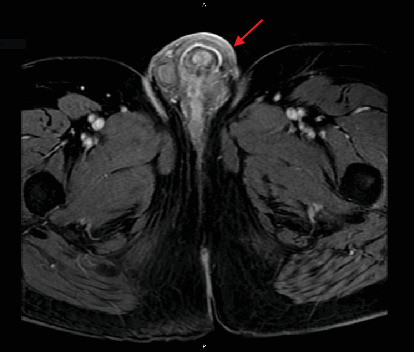
Magnetic resonance imaging (MRI) of the pelvis showing a heterogeneously enhancing lesion measuring 3.7 by 3.6 cm near the left penile base (indicated by the red arrow).

A 67-year-old African-American male with a past medical history of HIV managed with highly active antiretroviral therapy, benign prostatic hyperplasia, erectile dysfunction, and recurrent urethral stricture disease presented with a 1-month history of voiding symptoms associated with minimal pain. The examination was notable for a firm, nontender nodularity near the left base of the penis without associated skin changes. Cystoscopy revealed dense bulbar and proximal penile urethral stricture near the palpable penile mass. Magnetic resonance imaging (MRI) of the pelvis revealed a heterogeneously enhancing lesion measuring 3.7 by 3.6 cm near the left penile base (Figure 1). The patient was then taken to the operating room for excisional biopsy of the penile mass, which grossly measured 0.7 × 0.6 × 0.3 cm.

**Figure 2:**
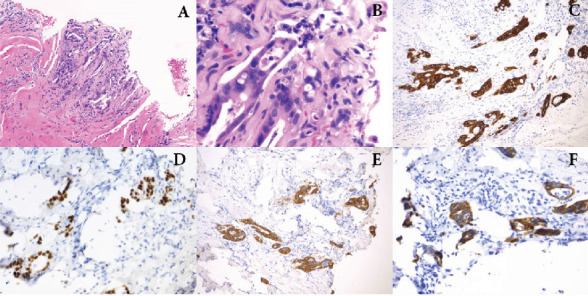
A small focus of malignant cells forming atypical glands, infiltrating the corpora tissue in an inflamed background, (A) H&E ×4 and (B) H&E ×40. Malignant cells are strongly positive for CK20 (C), CDX-2 (D), and pancytokeratin (E) and positive for CK7 (F) ×10. These findings are concerning for carcinoma of colorectal origin.

Histopathological analysis of the lesion revealed characteristics of invasive adenocarcinoma, and immunohistochemical testing was compatible with metastatic colorectal adenocarcinoma. Specifically, the tissue was positive for pancytokeratin, CK20, CDX-2, and CK7, and negative for GATA-3, p63, CMV, GMS, and HSV2 virus (Figure 2).

Further diagnostic testing was notable for elevations in serum tumor markers CA 19-9 (127.6 U/mL, normal 0.6–55.0 U/mL) and carcinoembryonic antigen (33.05 ng/mL, normal 0–3 ng/mL) and whole body PET/CT scan revealed multiple subcentimeter pulmonary nodules, but no colonic abnormalities. Hypermetabolic activity was noted near the base of the penis and in several subcentimeter inguinal lymph nodes bilaterally. A colonoscopy was performed, showing several areas of ulceration and focally congested mucosa within the cecum and rectum, respectively; however, the biopsy of these sites was negative for malignancy. No apparent polyps or other lesions were identified. Genomic analysis with Guardant360 (Guardant Health, Redwood City, CA) and FoundationOne (Foundation Medicine, Cambridge, MA) revealed mutations in *KRAS*, *SMAD4*, and *TP53*, with equivocal microsatellite instability and indeterminate tumor mutational burden.

Despite the absence of a primary colorectal lesion, the patient was started on chemotherapy with a modified FOLFOX-6 regimen (mFOLFOX-6; oxaliplatin with 5-fluorouracil and folinic acid) and has since completed treatment in full. Of note, the patient experienced refractory upper extremity neuropathy, and oxaliplatin dose was adjusted accordingly.

Follow-up CT imaging prior to the 12th cycle revealed the persistence of previously noted hypermetabolic activity in several subcentimeter inguinal lymph nodes bilaterally, as well as prominent bilateral axillary lymph nodes with FDG avidity and two foci of FDG avidity within the penis likely corresponding to metastatic disease. Given this concern for residual disease, the patient is planned to initiate radiation therapy to the pelvis.

## 3. Discussion

Secondary penile cancer of any origin is a rare phenomenon. Most cases of colorectal adenocarcinoma are accompanied by metastases to the lungs, liver, and/or pelvis and appear years after a successful initial treatment [1–3]. The prognosis for metastatic colorectal adenocarcinoma is generally poor, with a median overall survival ranging from 11 to 30 months depending on the tumor's profile [11, 12]. However, due to the rarity of secondary penile cancers, particularly those originating from colorectal cancer, survival outcomes are less well-defined. For metastatic cancer to the penis, the average overall survival is shorter, ranging from 9 to 14 months [5, 13, 14]. Further, most penile metastases from colorectal magnicides stem specifically from rectal carcinoma, while metastasis from colonic masses is less frequent, with only 10 reported cases since 1952 [3, 8, 15].

Although rare, prior case reports of colorectal metastases to the penis have been documented [4]. The majority of these cases involve patients with a known history of previously diagnosed and/or treated colorectal carcinoma with now metachronous penile metastases, often with multiple other sites of metastases [4, 8, 16–20]. However, in fewer instances, penile metastasis has been reported as the initial presenting symptom of metastatic colorectal cancer, often occurring alongside other systemic metastases and with an identifiable primary malignancy on workup [4, 9, 10, 21].

Here, we present a unique case of an isolated penile metastasis as the initial symptom of colon adenocarcinoma without a history of previous malignancy. Not only is an isolated penile metastasis as the initial presenting symptom of colorectal cancer an extremely rare manifestation, but to our knowledge, this is also the first such reported case in the literature without an identifiable primary colonic mass.

## 4. Proposed Theories of Dissemination

Given its rarity, the mechanism of penile metastasis has yet to be elucidated. In 1956, Paquin and Roland posited numerous theories including retrograde venous, lymphatic, or arterial spread, and, less likely, direct growth of tumor into adjacent organs or implantation [22]. Among these, retrograde venous flow is thought to represent the most likely mechanism [1, 2, 22].

Specifically, blood from the cavernous spaces of the penis drains through vessels that converge into the deep dorsal vein or pass through the base of the penis to join the prostatic plexus [1, 2, 18]. These venous networks communicate with the pudendal, vesical, vertebral, and hemorrhoidal plexuses, allowing for a connection between the dorsal venous system of the penis and venous plexuses of the pelvic organs. Consequently, a reversal of blood flow, possibly by tumor obstruction, could lead to tumor spread to the penis [1, 2, 18]. In fact, such retrograde flow may explain why most secondary penile lesions affect the corpora cavernosa and glans [1]. Conversely, metastasis to the penis via arterial spread is likely uncommon, possibly occurring through the internal pudendal and iliac arteries, either by direct tumor extension into the arterial pathways or from metastatic tumor emboli [1–4, 22].

Another commonly proposed theory involves retrograde lymphatic spread, which occurs similarly to retrograde venous flow [1, 22]. The superficial inguinal nodes drain the skin of the penis, while lymphatics from the glans and corpora cavernosa drain into either superficial or deep nodes. In the pelvic region, the lower rectum drains through the perineum into the inguinal and iliac nodes [1, 2, 18]. The proximity of these lymphatic networks allows for a potential connection between the penile and pelvic regions and may explain resulting secondary lesions to the penile skin [2].

## 5. Detection and Treatment

Secondary penile cancer varies in presentation, but 60% report symptoms related to the presence of nodules which often involve both corpora cavernosa [1, 2]. These nodular symptoms include lower urinary tract symptoms, priapism, skin lesions, and penile and/or perineal pain in advanced cases [1–5]. In our case, the tumor presented as a unilateral mass and urethral stricture with urinary symptoms. As these symptoms are somewhat nonspecific, the etiology must be clarified in order to differentiate malignancy from benign penile lesions, infectious causes, and other benign conditions such as Peyronie's disease [1–3].

Biopsy or corporal aspiration is the standard of diagnosis to differentiate between metastasis and primary penile cancer [1, 2]. In the case of penile metastasis, a pathological assessment with immunohistochemistry is integral to determining the primary site, particularly in cases with no prior history of malignancy or an identifiable primary lesion [3, 8]. Supplementary imaging includes ultrasonography, CT, and/or MRI for staging and detection of other metastatic sites, and adjunctive genomic testing can be performed to identify mutational profiles for precision medicine [1, 2, 23].

The management of penile metastasis is dependent on the site of the primary lesion, the degree of additional metastatic burden, and the severity of symptoms [2]. As the presence of a penile metastasis typically underlies advanced or end-stage primary disease with a poor prognosis, treatment is often palliative. Treatment modalities include chemotherapy, radiotherapy, palliative care, and surgical excision including penectomy [4]. In this case, the patient underwent therapeutic systemic chemotherapy with mFOLFOX-6, with successful resolution of his urinary symptoms, though the patient did experience persistent neuropathy of the hands, a common side effect of oxaliplatin [24].

Unlike most previously reported cases, our patient had no symptoms of colorectal disease, no identifiable primary colorectal lesion, and no other identifiable metastatic lesions. Our patient also lacked significant penile or cutaneous involvement that would warrant aggressive surgical treatment and was instead managed with excisional biopsy followed by systemic chemotherapy and pelvic radiotherapy. Still, such an initial presentation may suggest a more favorable prognosis than has previously been described. Close surveillance via serum tumor markers (CEA and CA 19-9), cross-sectional imaging, and colonoscopy will be imperative as treatment continues.

## 6. Conclusion

Although rare, secondary penile cancer should be considered in patients with relevant genitourinary symptoms, particularly in cases with a history of underlying malignancy. Here, we describe a rare case of isolated penile metastasis as the initial manifestation of colorectal adenocarcinoma with no identifiable primary mass. While current literature suggests poor prognosis and palliative management for penile metastasis, the unique and relatively indolent presentation of this patient may portend a more favorable outcome, although optimal treatment and accurate prognosis remain unclear.

## Data Availability

The data that support the findings of this study are available from the corresponding author upon reasonable request.

## References

[1] Mearini L., Colella R., Zucchi A., Nunzi E., Porrozzi C., Porena M. (2012). A Review of Penile Metastasis. *Oncology Reviews*.

[2] Cherian J., Rajan S., Thwaini A., Elmasry Y., Shah T., Puri R. (2006). Secondary Penile Tumours Revisited. *International Seminars in Surgical Oncology*.

[3] Yin G. L., Zhu J. B., Fu C. L., Ding R. L., Zhang J. M., Lin Q. (2022). Metachronous Isolated Penile Metastasis From Sigmoid Colon Adenocarcinoma: A Case Report. *World Journal of Clinical Cases*.

[4] Kuliavas J., Dulskas A., Drachneris J., Miseikyte-Kaubriene E., Samalavicius N. E. (2018). Penile Metastasis From Rectal Carcinoma: Case Report and Review of the Literature. *Visceral Medicine*.

[5] Dong Z., Qin C., Zhang Q. (2015). Penile Metastasis of Sigmoid Colon Carcinoma: A Rare Case Report. *BMC Urology*.

[6] Lotfollahzadeh S., Recio-Boiles A., Cagir B. (2023). *Colon Cancer*.

[7] Riihimaki M., Hemminki A., Sundquist J., Hemminki K. (2016). Patterns of Metastasis in Colon and Rectal Cancer. *Scientific Reports*.

[8] Triki W., Kacem A., Itami A., Baraket O., Rebai M. H., Bouchoucha S. (2019). Penile Metastasis of Colon Carcinoma: A Rare Case Report. *Urology Case Reports*.

[9] Banerjee G. K., Lim K. P., Cohen N. P. (2002). Penile Metastasis: An Unusual Presentation of Metastatic Colonic Cancer. *Journal of the Royal College of Surgeons of Edinburgh*.

[10] Ferhi K., Ferhi A., Oussedik K., Bensallah K., Sibert L. (2006). A Penile Metastasis as the First Manifestation of Colon Cancer. *Journal de Chirurgie*.

[11] Biller L. H., Schrag D. (2021). Diagnosis and Treatment of Metastatic Colorectal Cancer. *Journal of the American Medical Association*.

[12] Modest D. P., Ricard I., Heinemann V. (2016). Outcome According to KRAS-, NRAS- and BRAF-Mutation as Well as KRAS Mutation Variants: Pooled Analysis of Five Randomized Trials in Metastatic Colorectal Cancer by the AIO Colorectal Cancer Study Group. *Annals of Oncology*.

[13] Lin Y. H., Kim J. J., Stein N. B., Khera M. (2011). Malignant Priapism Secondary to Metastatic Prostate Cancer: A Case Report and Review of Literature. *Reviews in Urology*.

[14] Cocci A., Hakenberg O. W., Cai T. (2018). Prognosis of Men With Penile Metastasis and Malignant Priapism: A Systematic Review. *Oncotarget*.

[15] Powell B. L., Craig J. B., Muss H. B. (1985). Secondary Malignancies of the Penis and Epididymis: A Case Report and Review of the Literature. *Journal of Clinical Oncology*.

[16] Schroeder T., Plambeck B., Bowdino C., DiMaio D., Christiansen A. (2021). Metastasis of Rectal Adenocarcinoma to the Penis and Scrotum in an Adult. *Cureus*.

[17] Persec Z., Persec J., Sovic T., Rako D., Savic I., Marinic D. K. (2014). Penile Metastases of Rectal Adenocarcinoma. *Journal of Visceral Surgery*.

[18] Nasrallah O. G., Fawaz M. W., Mahdi J. H., Armache A. K., El Sayegh N., Nasr R. W. (2024). Peno-Scrotal Metastasis of Colorectal Adenocarcinoma: A Case Report and Review of the Literature. *International Journal of Surgery Case Reports*.

[19] Efared B., Ebang G. A., Tahirou S. (2017). Penile Metastasis From Rectal Adenocarcinoma: A Case Report. *BMC Research Notes*.

[20] Kazama S., Kitayama J., Sunami E. (2014). Urethral Metastasis From a Sigmoid Colon Carcinoma: A Quite Rare Case Report and Review of the Literature. *BMC Surgery*.

[21] Cholin L., Perz S., Mahmood F., Zafar S. (2015). Palpable Penile Metastases: A Bizarre Presentation of Rectal Adenocarcinoma. *Case Reports in Urology*.

[22] Paquin A. J., Roland S. I. (1956). Secondary Carcinoma of the Penis. A Review of the Literature and a Report of Nine New Cases. *Cancer*.

[23] Gupta R., Othman T., Chen C., Sandhu J., Ouyang C., Fakih M. (2020). Guardant360 Circulating Tumor DNA Assay Is Concordant With FoundationOne Next-Generation Sequencing in Detecting Actionable Driver Mutations in Anti-EGFR Naive Metastatic Colorectal Cancer. *Oncologist*.

[24] Pachman D. R., Qin R., Seisler D. K. (2015). Clinical Course of Oxaliplatin-Induced Neuropathy: Results From the Randomized Phase III Trial N08CB (Alliance). *Journal of Clinical Oncology*.

